# Dose Responsive Effects of Subcutaneous Pentosan Polysulfate Injection in Mucopolysaccharidosis Type VI Rats and Comparison to Oral Treatment

**DOI:** 10.1371/journal.pone.0100882

**Published:** 2014-06-25

**Authors:** Michael Frohbergh, Yi Ge, Fanli Meng, Nesrin Karabul, Alexander Solyom, Alon Lai, James Iatridis, Edward H. Schuchman, Calogera M. Simonaro

**Affiliations:** 1 Genetics and Genomic Sciences, Icahn School of Medicine at Mount Sinai, New York, New York, United States of America; 2 Orthopedics, Icahn School of Medicine at Mount Sinai, New York, New York, United States of America; 3 Department of Pediatrics, University of Mainz, Mainz, Germany; 4 Department of Pediatrics, University of Pécs, Pécs, Hungary; Baylor Research Institute, United States of America

## Abstract

**Background:**

We previously demonstrated the benefits of daily, oral pentosan polysulfate (PPS) treatment in a rat model of mucopolysaccharidosis (MPS) type VI. Herein we compare these effects to once weekly, subcutaneous (sc) injection. The bioavailability of injected PPS is greater than oral, suggesting better delivery to difficult tissues such as bone and cartilage. Injected PPS also effectively treats osteoarthritis in animals, and has shown success in osteoarthritis patients.

**Methodology/Principal Findings:**

One-month-old MPS VI rats were given once weekly sc injections of PPS (1, 2 and 4 mg/kg, human equivalent dose (HED)), or daily oral PPS (4 mg/kg HED) for 6 months. Serum inflammatory markers and total glycosaminoglycans (GAGs) were measured, as were several histological, morphological and functional endpoints. Overall, weekly sc PPS injections led to similar or greater therapeutic effects as daily oral administration. Common findings between the two treatment approaches included reduced serum inflammatory markers, improved dentition and skull lengths, reduced tracheal deformities, and improved mobility. Enhanced effects of sc treatment included GAG reduction in urine and tissues, greater endurance on a rotarod, and better improvements in articular cartilage and bone in some dose groups. Optimal therapeutic effects were observed at 2 mg/kg, sc. No drug-related increases in liver enzymes, coagulation factor abnormalities or other adverse effects were identified following 6 months of sc PPS administration.

**Conclusions:**

Once weekly sc administration of PPS in MPS VI rats led to equal or better therapeutic effects than daily oral administration, including a surprising reduction in urine and tissue GAGs. No adverse effects from sc PPS administration were observed over the 6-month study period.

## Introduction

The mucopolysaccharidoses (MPS) comprise a group of 11 lysosomal storage disorders due to inherited deficiencies in enzymes required to degrade glycosaminoglycans (GAGs) [1]–[3]. The specific features of each MPS type are associated with the type of GAG that accumulates in the cells and tissues of affected individuals. The majority of MPS patients share debilitating skeletal deformities, including dysostosis multiplex, reduced bone growth, joint and spine degeneration, contractures, vertebral fusions, tracheal deformities, chest deformities and others. Cardiac, pulmonary and neurological abnormalities also may occur. Currently, treatment for MPS includes hematopoietic stem cell transplantation (HSCT) using donor bone marrow or cord blood cells, and/or enzyme replacement therapy (ERT) [4]–[7]. ERTs are currently approved for four MPS types (I, II, IV and VI), and are under development for others. Overall, the effectiveness of both HSCT and ERT on the skeletal and connective tissue manifestations of MPS is limited, presenting a need for improved and/or alternative treatments [8]–[10].

Our laboratory has used animal models of MPS to investigate the pathogenic mechanisms of cartilage/bone disease in these disorders, with the long-term goal of developing such alternative therapies [11]–[14]. In summary, we have found that GAG storage in MPS activates the toll-like receptor 4 (TLR4) signaling pathway, leading to the release of tumor necrosis factor (TNF)-α and other inflammatory cytokines, and resulting in downstream tissue damage including cell death [12]. These changes occur in all MPS types and are not restricted to specific GAG accumulation. They also occur in all MPS cells, but are most evident in cells with abundant GAG storage such as chondrocytes [14]. Based on these findings, we proposed that treatment of MPS with anti-inflammatory drugs that target TNF-α and/or other inflammatory pathways activated in MPS would be beneficial, alone or in conjunction with other therapies. Proof-of-principal was obtained using MPS VII/TLR4 double knockout mice, and by treatment of MPS VI rats with anti-TNF-α antibodies [12].

We next evaluated pentosan polysulfate (PPS) for the treatment of MPS. PPS is a low molecular weight, carbohydrate polymer isolated from beech trees [15]. It is manufactured in both oral and injectable forms, and has been available for over 40 years as an anti-thrombotic agent [16], [17]. Subsequent work identified the anti-inflammatory properties of PPS, leading to its use as an oral treatment for interstitial cystitis (IC, painful bladder syndrome; Elmiron) [18], [19]. Injectable PPS forms also are used to treat osteoarthritis in veterinary medicine (e.g., Cartophen Vet) [20], [21]. The bioavailability of the injectable form is greater than oral [personal communication – bene pharmaChem, Germany], with the majority of the drug targeted to the liver and kidney. Despite the weak anti-thrombotic properties of PPS, long-term treatment with Elmiron has an excellent safety profile in IC patients.

Due to the i) potent anti-inflammatory properties of PPS, including the positive effects observed in animals with arthritis, ii) positive and extensive long-term human safety data, iii) availability in various formulations and approval by several regulatory authorities, and iv) cost-effectiveness compared to other MPS therapies, including ERTs, we undertook a study to evaluate PPS treatment for MPS. Rats with MPS VI were treated with daily, oral PPS by gavage (4 mg/kg, human equivalent dose (HED) [22]), leading to a marked reduction in inflammation, both systemically and locally in the joints, resulting in improvements in motility, cranial lengths, tracheal deformities, dentition, including tooth mineral densities, and other characteristic features of this MPS model [11].

The current study was designed to compare these findings to once weekly subcutaneous (sc) injections of PPS in the same animal model, and to document the dose responsive effects. The rationale underlying sc PPS injections is based on the enhanced bioavailability of the injected drug, as well as potentially better compliance and cost-effectiveness of a once weekly injection compared with daily oral administration. Further, for animal studies sc administration provides more consistent dosing than treatment by gavage. Three different weekly sc doses were evaluated (1, 2 and 4 mg/kg, HED) and compared to daily oral treatment (4 mg/kg, HED).

## Materials and Methods

### Animals

The MPS VI rat model has been previously described and used extensively [12]–[14]. A breeding colony was established from heterozygous mating pairs, and genotyping was performed on tail clip DNA using established methods [23]. Euthanasia of rats was performed using carbon dioxide inhalation. All animal protocols were approved by the Mount Sinai Institutional Animal Care and Use Committee (protocol # 08-0108), and were performed in accordance with NIH guidelines.

### Treatment of MPS VI Rats with PPS

Powdered PPS was obtained from bene pharmaChem (Germany) and freshly dissolved in sterile saline before each use. Three week-old female MPS VI rats were randomized into three groups and received weekly sc PPS injections at 1, 2 and 4 mg/kg (HED, n = 6/group). A fourth group (n = 6) was treated by gavage using PPS added to the drinking water (4 mg/kg, HED), according to our previous methods [11]. Age and gender-matched untreated MPS VI and normal rats were maintained as controls (n = 6/group). All experimental rats were treated until they reached 7 months of age. Only female rats were used to avoid gender-specific differences. Studies were coded at the time of drug administration so as the evaluators were not aware of the conditions being observed and a blind study was implemented. At the end of the study the animals were euthanized and tissues were collected from the control and treated MPS VI rats and fixed in neutral buffered 10% formalin (Sigma Chemical, St. Louis, MO) for histology and microCT analysis.

### Serum Immunoassays

Serum was collected monthly from the control and treated rats, and inflammatory cytokines were assessed by enzyme-linked immunosorbent assays (ELISAs) using rat ELISA kits according to the manufacturers' protocols. For rat TNF-α and TNF receptor 1 (TNFR1), catalog #RTA00 and MRT10 from R & D Systems (Minneapolis, MN) were used, respectively; for interleukin-8 (IL-8) catalog #R6417 from Biotang Inc. (Waltham, MA); for the S100A8/A9 complex catalog #MBS036705 from MyBiosource.com (San Diego CA); and for C-reactive protein (CRP) catalog #RAB0097 from Sigma-Aldrich Inc. (St. Louis, MO). All serum assays were performed in triplicate.

### Total GAG Assays

Tissues (liver, spleen and kidney) were collected and homogenized according to the BLYSCAN GAG kit #NC0287381 (Fisher Scientific, Walthman, MA). Total urine GAGs also were measured using this kit followed by creatinine assays using the Microvue creatinine assay kit catalog #NC9732564 (Fisher Scientific, Walthman, MA).

### Growth Plate and Articular Cartilage Histology

H & E staining was used to observe cellular differences in the growth plates and articular cartilage of treated samples. Whole knees were removed from the specimens and decalcified for histology preparation. The samples were cut in half down the center of the trochlea and embedded in paraffin for sectioning. Routine H & E staining was performed to see chondrocyte orientation and morphology in both the growth plate and the articular surface of the trochlea.

### Radiographic and MicroCT Image Acquisition

Femoral, tibial, humeral, radial, skull and snout lengths were measured using x-ray radiographs. Animals were anesthetized using 250 mg/kg IP injection of Avertin (Alfa Aesar, Ward Hill, MA) and full body x-rays were taken using a 43085 X-ray system (Faxitron Series, Hewlett Packard, Palo Alto, CA). The animals were positioned on their stomach and on their sides and imaged at 32 kV for 10 seconds. Films were developed in an M35A X-Omat Processor (Kodak, Rochester, NY). Films with the animal oriented on its stomach were used to measure femoral, tibial, humeral and radial lengths. The films with side orientation were used to measure skull and snout lengths.

For microCT imaging, samples were prepared by removal of soft tissue and fixation in 10% formalin. All samples were scanned on a Skyscan 1172 X-ray microtomography (microCT) system (Micro Photonics, Allentown, PA). Samples were held in place by homemade fixtures mounted to the interior stand and wrapped in Parafilm to prevent the sample from dehydrating. To image the skulls, the instrument parameters were set to 70 kV, 142 µA and a 26.7 pixel size resolution. For femurs and spines, the parameters were 59 kV and 169 µA with 9.1 pixel resolution for the femurs and 15 pixel resolution for spines. For all, a 0.5% aluminum filter was applied with a rotation step of 0.5, a frame average of 6 and 180° rotation.

3D reconstruction of all samples was done using the NRECON Reconstruction software (Micro Photonics, Allentown, PA) using a ring artifact correction of 10. For skulls, a beam hardening correction (%) of 40 and a threshold scale of 0–0.1 was used. For femurs and spines, a 60% beam hardening correction was used. Morphological analysis was performed on reconstructed images analyzed using Ct Analyzer (CtAn) and Data Viewer (Micro Photonics, Allentown, PA), and 3D images were generated using the CtVox (Micro Photonics, Allentown, PA) software. Trabecular and cortical bone values were generated using CtAn software. For trabecular analysis, percent bone volume (BV/TV), bone surface volume ratio (BS/BV), trabecular thickness (Tb.Th), trabecular separation (Tb.Sp), trabecular number (Tb.N) and total porosity (Po(tot)) were recorded. For cortical analysis, total cross-sectional area (Tt.Ar), cortical bone area (Ct.Ar), medullary area (Ma.Ar), cortical area fraction (Ct.Ar/Tt.Ar), cortical thickness (Ct.Th), total perimeter (T.Pm), periosteal perimeter (Ps.Pm), endosteal perimeter (Ec.Pm) and total porosity (Po(tot)) were recorded. Bone mineral density (BMD) and tissue mineral density (TMD) were calculated using “phantom” samples of calcium hydroxyapatite (CaHA) with known densities of 0.25 g/cm^3^ and 0.75 g/cm^3^ to establish attenuation coefficients to generate upper and lower thresholds. Varying ranges of threshold were calculated based on resolution of the sample taken. For the fermurs, skulls and spines, the lower attenuated thresholds (0.25 g/cm^2^) were 0.01581, 0.1578, and 0.01801, respectively. The higher attenuated thresholds (0.75 g/cm^2^) were 0.03973, 0.3556, and 0.04334, respectively. These values were then normalized to a lower threshold of 0 and an upper threshold of 1, and used as experimental values to obtain the BMD values. For tissue and tooth mineral density and cortical analysis, the same procedure was followed as for BMD, with the grayscale threshold set at 85–255.

#### Vertebrae

The T11-L2 region of the spinal column was excised from treated and control rats. The vertebral columns were kept intact and imaged as a whole in order to assess morphological differences between vertebrae. Trabecular analysis and BMD measurements were performed on the vertebral body of the L2 vertebrae.

#### Femur

Whole femurs were excised from age and gender matched rats. Due to variances in femoral length, the typical method of measurement analysis had to be adjusted. For cortical analysis of normal bones, the top region of interest (ROI) was measured 9 mm distally from the apex of the growth plate (appearance of the metaphysis) and a 2 mm section of the diaphysis was analyzed. For MPS VI animals with significantly shorter femurs, the top ROI was measured 7.5 mm distally from the apex of the growth plate and a 1 mm section of the diaphysis was analyzed. The changes in measurements were compensated by similar positioning at the middle of the diaphysis. For trabecular analysis, top ROI's were chosen by observing the images until the metaphysis was no longer viewed, and then using a 1 mm section measured distally from the top ROI. Reconstructed images were used in order to assess morphological differences between the growth plates and the trabeculae at the distal end of the femur.

#### Skulls

Whole skulls were excised from treated and control rats. Morphological analysis and size characterization was performed to observe significant differences in whole skull and snout lengths as well as molar misalignment and lower incisor overgrowth. Tooth mineral density was performed on the lower incisors from the anterior tip of the teeth until the presence of the mandibular bone was observed.

### Three-Point Bending Tests

Three point-bending flexural tests were performed using an Instron (model 8872) material testing system (Instron, Norwood, MA) equipped with Labview software for analysis. Whole femurs were excised from all control and treated groups and all samples were tested until failure. Data was collected at 200 Hz at a load of 50N/V and a single ramp waveform at 1 mm/s down to 10 mm. A working distance of 17 mm was used for all samples. All soft tissue was removed from the bones and samples were stored in −80°C until ready for testing. Thawed samples were placed onto a homemade 3-point flex apparatus where the femoral epiphyses were positioned outside of the testing area and the ends of the midshaft were positioned inside the testing area. The force was applied at the center of the midshaft in the anteroposterior direction. An initial load of 1.0N was applied prior to beginning the test. Force and deflection were converted to stress and strain (Equations 1 and 2, respectively) to determine flexural modulus and ultimate flexural strength, where σ is stress, ε is strain, F is force, L is the working distance, R is radius, H deflection, and h is height: 1) σ = FL/πR^2^, and 2) ε = 6 Hh/L^2^. Bone geometry was estimated to cylindrical for stress/strain conversions. Stiffness and force at failure also were recorded as values of whole bone mechanics by plotting applied force vs. deflection.

### Rotarod Analysis

All rats were assessed and compared at the end of the study, (7 months of age). Rats were primed on the rod for 2 consecutive days prior to the actual recording. The rotarod was set at increasing speeds from 10 to 40 rpm over 3 minutes, and an average of the latency to fall off from the rod was recorded. Results were analyzed by one-way analysis of variance (ANOVA) with the variable group. In addition, videos were taken of the rats to document appearance and mobility in open space.

### Biodistribution of Labeled PPS in MPS VI Rats

PPS-Rhodamine B was prepared with a degree of substitution per xylose of 0.0011 (provided by bene pharmaChem, Germany). Imaging work was performed at the Translational and Molecular Imaging Core facilities at the Icahn School of Medicine at Mt. Sinai (New York, NY) using the Spectrum Preclinical In Vivo Imaging System (IVIS) from Perkin Elmer. Age and sex-matched MPS VI rats received either an oral or sc injection of 10 mg/kg HED of the labeled PPS. The animals were sacrificed 24 hours post dose, tissues were collected and immediately imaged using the IVIS. The settings used were: excitation = 570 nm, emission = 620 nm, binning (HR) 4, FOV: 22.8 cm and exposure time of 40 seconds.

### Data Presentation and Statistical Analyses

Six rats were enrolled in each of six groups (3 sc PPS treatment groups; one oral PPS group; untreated MPS VI and normal). Some microCT and biomechanical analyses was performed on a subset of these animals (as indicated in the Results). In vitro assays (e.g., ELISAs, GAG assays) and rotarod analyses were performed on all of the rats and replicated at least 3 times. Histological analysis was performed on representative sections prepared from all animals. For statistical analyses the data between 2 groups were subjected to student's t-test analysis, one-way analysis of variance (ANOVA) with the variable group, multivariate analyses of variance (MANOVAs) followed by post hoc Bonferroni adjustments. The results were considered significant at P<0.05. Statistics were performed using Sigma Stat 3.1 (Systat Software).

## Results

### Subcutaneous and Oral PPS Treatment Reduces Systemic Inflammation in MPS VI Rats

**Figure 1:**
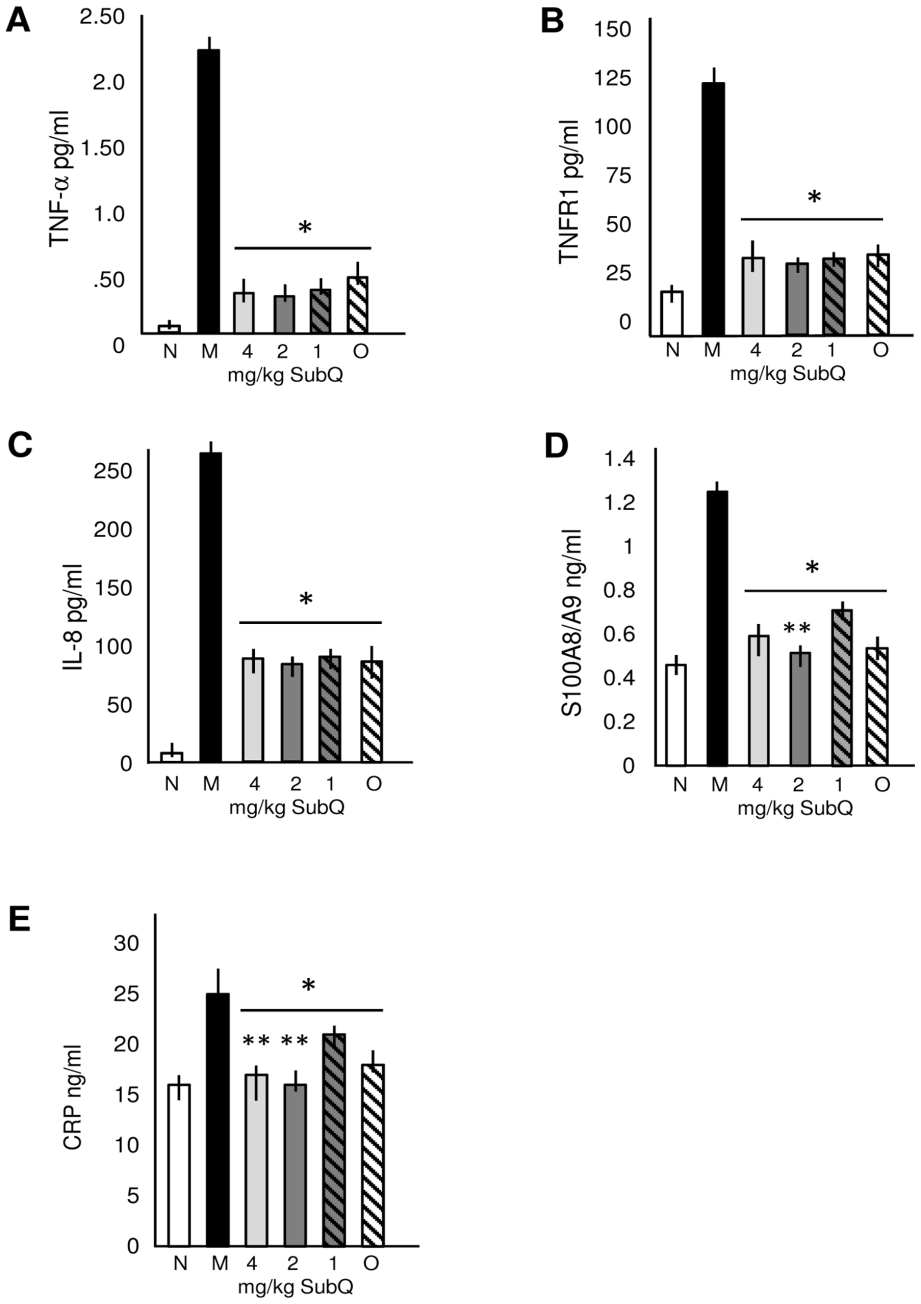
Serum inflammatory markers in normal, untreated and PPS treated MPS VI rats. Tumor necrosis factor-α (TNF-α (**A**), tumor necrosis factor receptor-1 (TNFR1) (**B**), interleukin-8 (IL-8) (**C**), protein S100A8/A9 (**D**), and C-reactive protein (CRP) (**E**), were quantified by ELISA assays as described in the Materials and Methods. White columns, normal rats; black columns, untreated MPS VI rats; light gray columns, 4 mg/kg sc PPS treated MPS VI rats; dark gray columns, 2 mg/kg sc PPS treated MPS VI rats; grey hatched columns, 1 mg/kg sc PPS treated MPS VI rats and white hatched columns, 4 mg/kg oral PPS treated MPS VI rats. N = 6/group. All doses are HED [22]. The vertical lines in each column indicate the ranges. * P<0.05 comparing treated to untreated MPS VI rats. **P<0.05 comparing sc PPS administration to oral.

Three groups of MPS VI rats were treated once weekly by sc injections of PPS (1, 2 and 4 mg/kg, HED) for 6 months beginning at ∼1 month of age. Another group was treated by daily oral gavage with 4 mg/kg (HED) according to our previous protocol [11]. By the end of treatment (7 months of age) all groups had markedly reduced levels of TNF-α, TNFR1, IL-8, S100 calcium protein A8/A9 complex (S100A8/A9) and CRP compared to untreated MPS VI controls (Figs. 1A–E). No significant differences were observed among the three sc dose groups, or between the oral vs. sc treatment groups. As was observed in our previous (oral) study [11], reduction of serum inflammatory markers was observed rapidly (within 1 month) of initiation of PPS treatment (data not shown).

### Growth Plate and Articular Cartilage Histology, and GAG Levels in MPS VI Rats

**Figure 2:**
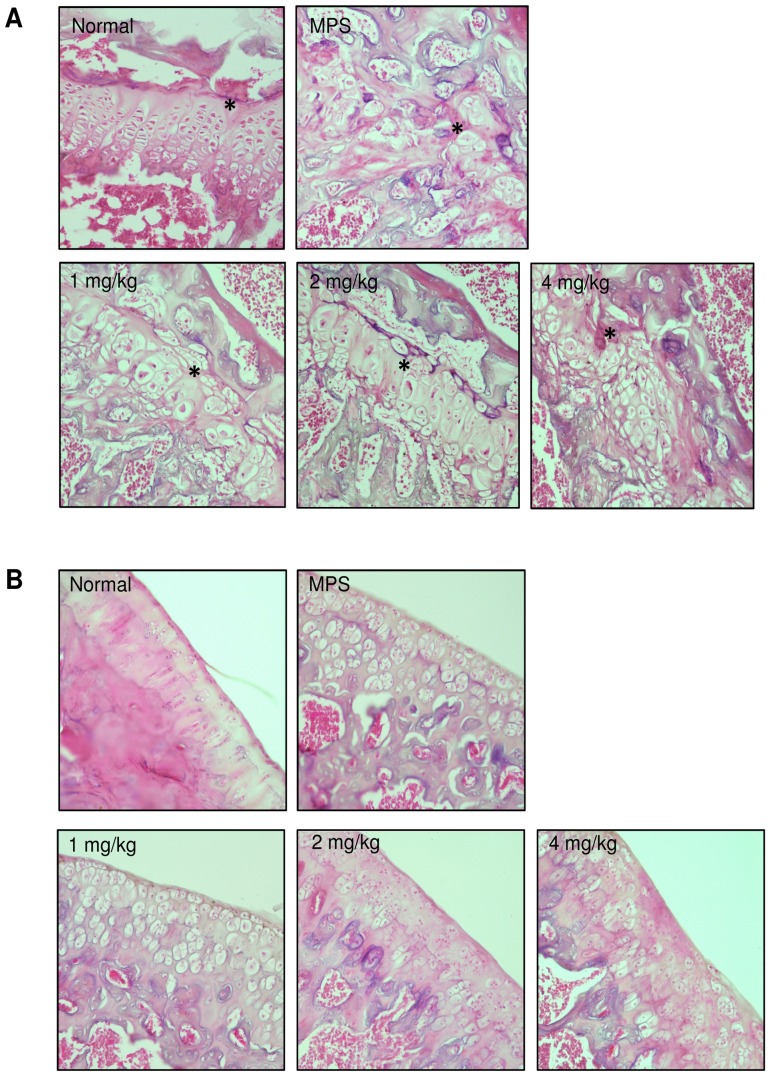
Histological analysis of MPS VI rat femoral growth plates and articular cartilage. Representative images are shown for untreated MPS VI rats and each of the sc treatment groups (H & E staining, 20× magnification). (**A**) Growth plate analyses. Seven-month-old MPS VI rats revealed a complete loss of columnar arrangement in the knee growth plates as compared to normal animals. Overall, PPS sc treatment resulted in moderately improved chondrocyte orientation and growth plate organization, although significant vacuolated chondrocytes were still observed. *Indicates the growth plate. (**B**) Articular cartilage analyses. Micrographs revealed that sc PPS treatment reduced vacuole formation, suggesting reduced GAG storage in MPS VI articular chondrocytes, in a dose dependent manner. Weekly sc injections of 1 mg/kg had little to no effect, while improvements can be seen at 2 and 4 mg/kg.

At the end of treatment rats were euthanized and their articular cartilage from the trochlear surface and femoral growth plates analyzed by histology (Fig. 2A and B). MPS growth plates contain vacuolated chondrocytes that are not organized into their normal columnar structure (Fig. 2A). As described previously, oral PPS treatment did not alter this phenotype [11]. In contrast, treatment with sc PPS led to modest reorganization of the femoral growth plate (Fig. 2A), particularly in the 2 mg/kg dose group, although this did not result in increased femur lengths (data not shown). Vacuolated chondrocytes also are evident in the articular cartilage of MPS VI rats, and sc PPS administration led to some improvements, including reduced vacuole size and chondrocyte orientation (Fig. 2B). We also observed improvements in the tracheal morphology of the sc treated animals, similar to what was observed previously by oral administration (Fig. S1).

**Figure 3:**
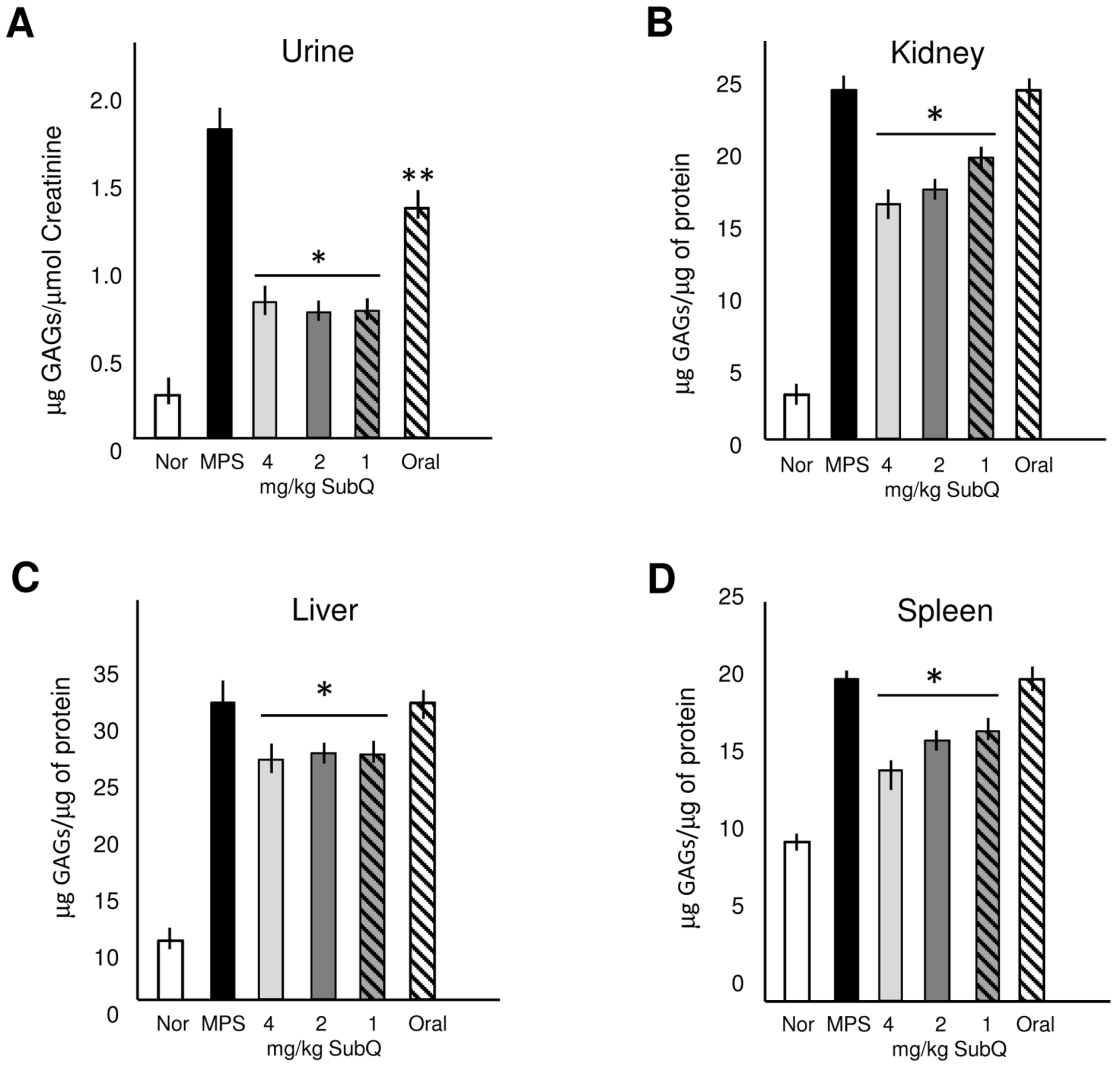
GAG reduction in PPS-treated MPS VI rats. (**A**) Total urine GAGs were significantly reduced in all PPS-treated MPS VI rat groups compared to untreated MPS control animals, regardless of the mode of administration. Subcutaneous treatment also significantly reduced urine GAGs compared to oral treatment. Tissue GAGs were significantly reduced only in the MPS VI rats treated with sc PPS. (**B**) kidney, (**C**) liver and (**D**) spleen. A dose responsive reduction was observed in the kidney and liver. *P<0.05 comparing treated to untreated MPS VI rats. ** P<0.05 comparing sc to oral treatment.

We next examined total GAG levels in the urine and tissue homogenates of the treated MPS VI rats (Fig. 3A–D). A reduction in urine GAGs was observed in all PPS treatment groups (oral and sc), although the reductions in the sc treated animals were significantly greater. Total tissue GAGs (liver, spleen, kidney) also were significantly reduced in the sc treatment groups, but not in the oral group. Dose responsive effects was observed in the spleen and kidney, but not in the urine and liver of the animals treated by sc PPS.

### MicroCT and Biomechanical Analysis of the Femurs and Vertebrae in PPS Treated MPS VI Rats

**Figure 4:**
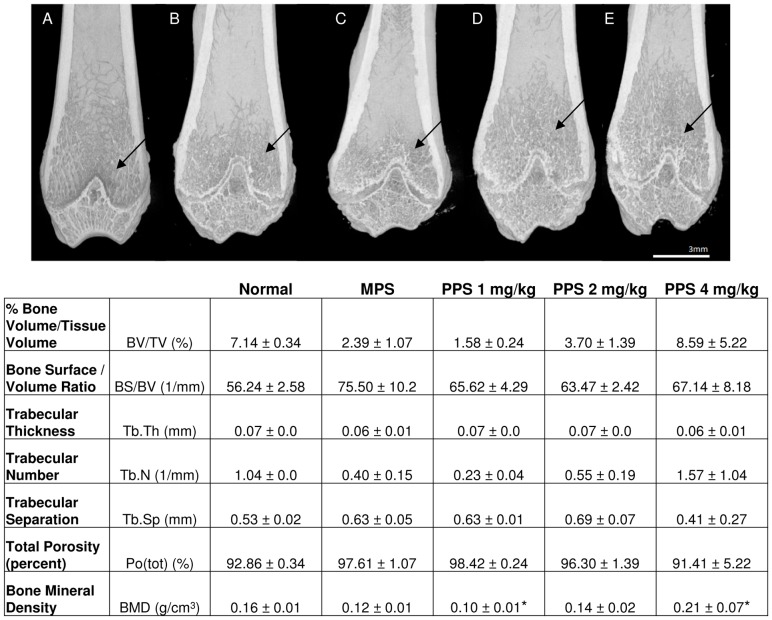
Femoral trabecular analysis of PPS-treated MPS VI rats. MicroCT analysis of the femurs was performed as described in the Materials and Methods. Representative images are shown for 7-month-old (**A**) normal, (**B**) untreated MPS VI, (**C**) 1 mg/kg, (**D**) 2 mg/kg, and (**E**) 4 mg/kg sc PPS-treated animals. Black arrows indicate the trabecular regions. MicroCT quantification of the trabeculae is shown below the images. Dose responsive improvements in several criteria were observed, including % bone volume/tissue volume (BV/TV), bone surface/volume ratio (BS/BV), trabecular number (Tb.N), trabecular separation (Tb.Sp), total porosity Po(tot), and bone mineral density (BMD). *indicates statistical differences between untreated and treatment groups (p<0.05).

MicroCT imaging was used to analyze the cortical and trabecular properties of the femurs, vertebrae and skulls of the sc treated MPS VI rats. MicroCT data already has been presented for oral treatment [11]. Fig. 4 summarizes the femoral data, and shows that untreated MPS VI animals exhibited reduced trabecular spreading up the shaft of the bone. This was improved in the 2 and 4 mg/kg dose groups. Improvements in % bone volume/tissue volume (BV/TV), bone surface/volume ratio (BS/BV), trabecular number (Tb.N), trabecular separation (Tb.Sp), total porosity [Po(tot)], and bone mineral density (BMD) also were observed, although most of these values did not reach statistical significance (other than BMD in the 1 and 4 mg/kg groups). However, a clear dose response was observed in several measurements (e.g., BV/TV, TbN, and BMD). No significant changes in cortical bone were observed between normal and MPS animals, which is consistent with the previously reported oral data [11] (Fig. S2).

**Figure 5:**
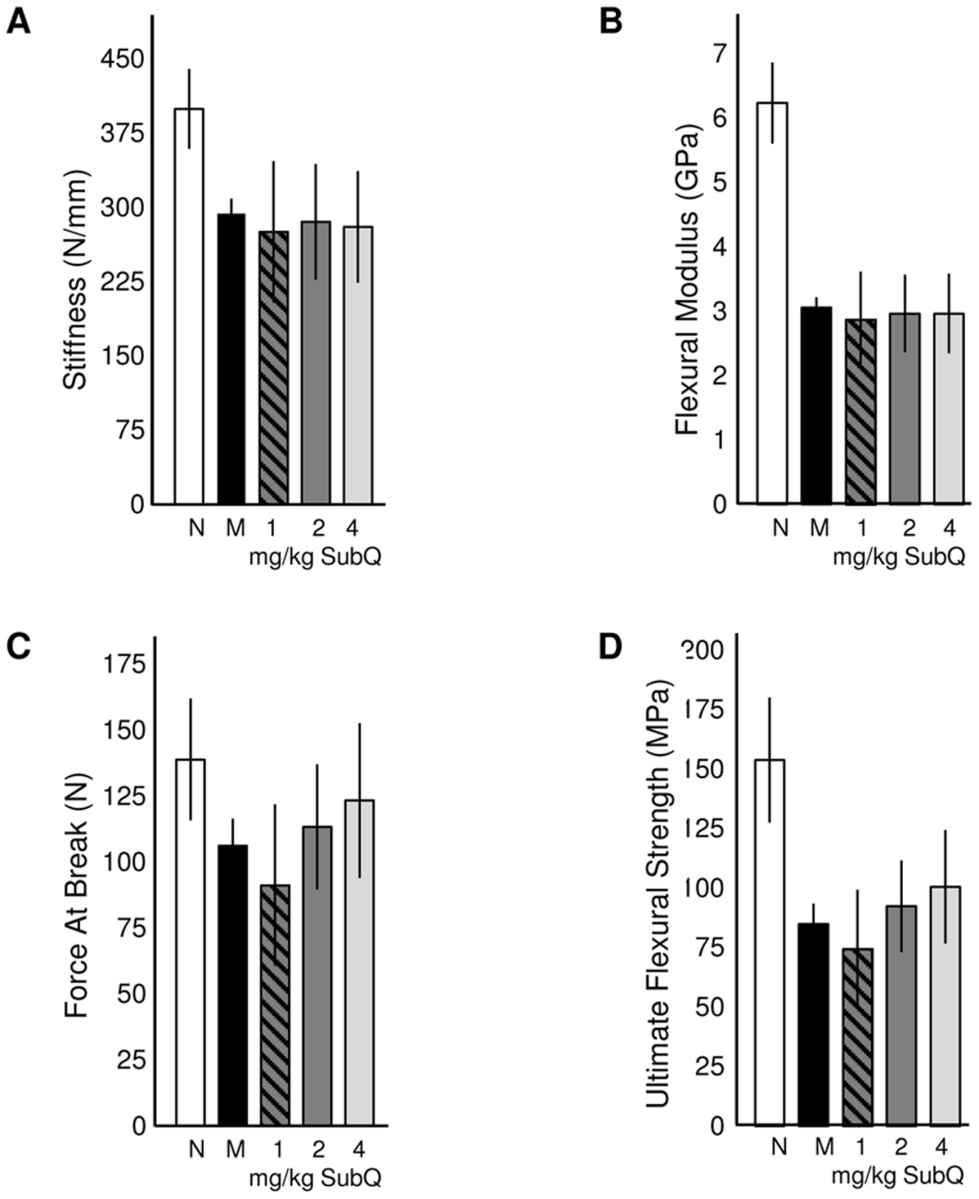
Flexural mechanics of normal, untreated MPS VI, and PPS-treated MPS VI femurs. The whole bone stiffness (**A**) and flexural modulus (**B**) were decreased in MPS VI compared to normal animals, and no changes were observed in the PPS treatment groups. However, force at break (**C**) and flexural strength (**D**) did reveal increasing improvements in a dose dependent manner, indicating that sc PPS treatment moderately enhanced the fracture strength of the femurs.

The biomechanical properties of the femurs were further analyzed by three-point bending analysis (Fig. 5). Overall, a decrease in the mechanical properties was observed in MPS VI animals when compared to normal. Treatment with sc PPS did not result in any changes in the mechanics at the tissue level, indicated by the stiffness and flexural modulus (Fig. 5A and B). However there was a clear dose dependent increase in the fracture force and ultimate flexural strength (Fig. 5C and D), indicating that whole bone mechanics were improved with treatment. No biomechanical improvements were observed from oral PPS treatment (data not shown).

**Figure 6:**
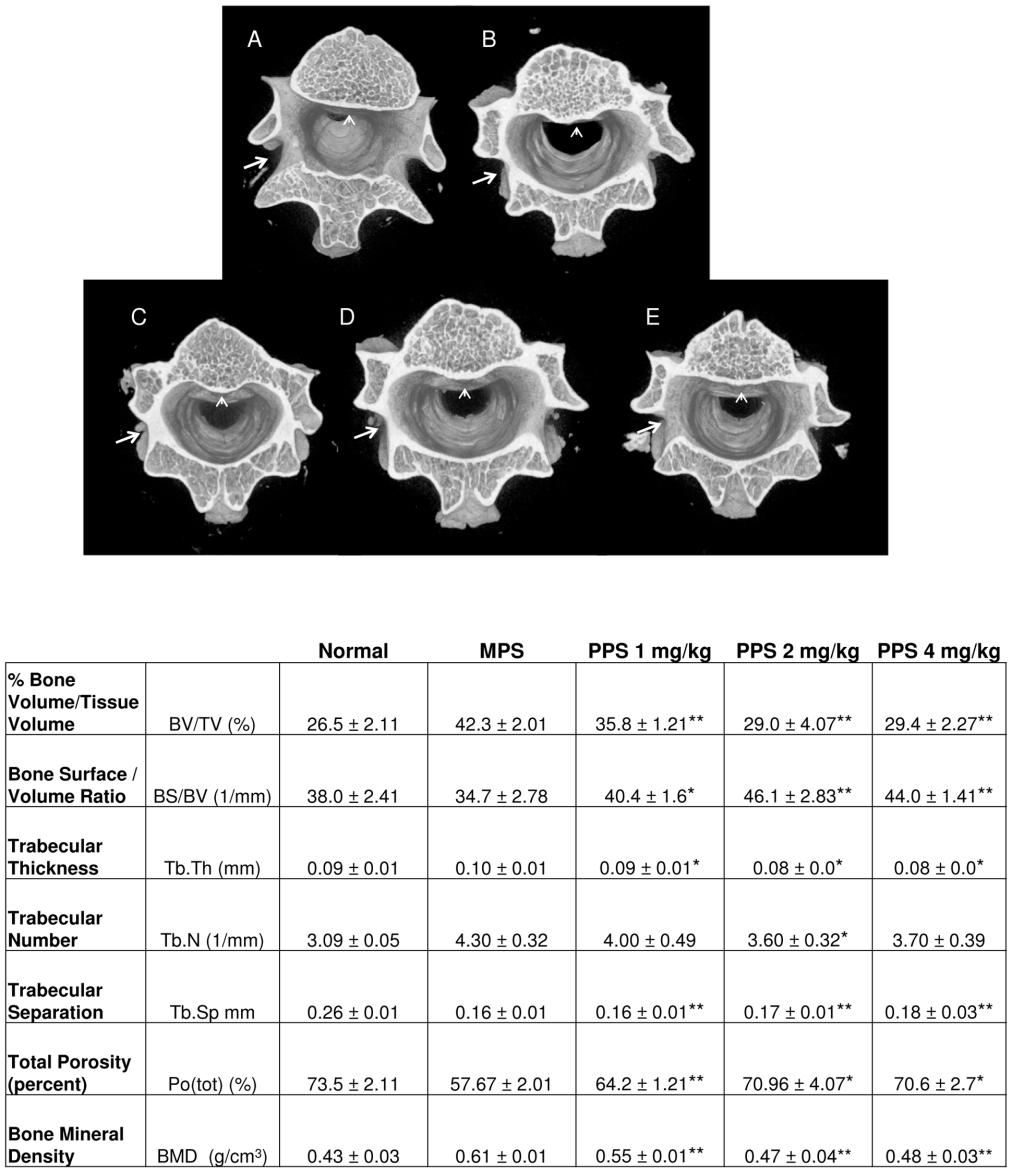
Spinal trabecular analysis of PPS-treated MPS VI rats. 3-D microCT reconstructions of (**A**) normal, (**B**) untreated MPS VI, (**C**) 1 mg/kg, (**D**) 2 mg/kg, and (**E**) 4 mg/kg sc treated T1 thoracic vertebrae. All images were taken at the point where the pedicle connected with the spinal body. As seen in the untreated MPS VI image (**B**), a significant amount of dense cortical tissue (indicated by a whiter color intensity) is present compared to the normal animal (**A**). The cortical tissue also appeared to grow into the trabecular area in MPS VI animals (indicated by arrows and arrowheads). The 2 and 4 mg/kg PPS doses reduced the cortical in-growth and promoted the formation of better-oriented trabeculae in the MPS VI rat vertebrae. P values to represent statistical differences are presented as * for p<0.05 and ** for p<0.01.

The vertebrae of the sc treated MPS VI rats also were analyzed by microCT (Fig. 6). In untreated MPS VI rat vertebrae substantial cortical overgrowth into the trabecular area was observed (indicated by arrows and arrowhead). This resulted in abnormal trabecular meshing within the vertebrae. These observations were correlated with elevated BV/TV values in MPS VI rats vs. normal, indicating higher amounts of bony, mineralized tissue in MPS. Opposite to femurs, in MPS VI rat vertebrae the trabecular numbers were increased compared to wild-type (i.e., they are reduced in MPS VI rat femurs). This also likely reflects the infiltration of cortical bone into the trabecular region of the vertebrae since the software cannot distinguish between cortical and trabecular tissue. Reduced porosity and increased BMD also is likely reflective of increased cortical bone. Subcutaneous PPS treatment resulted in significant improvements in most microCT measurements, and some clear dose responses were observed. The images also indicate that cortical overgrowths were reduced in the sc treated MPS VI rat vertebrae, most evident in the 4 mg/kg dose group.

Skull microCT revealed improved dentition, tooth mineral densities and snout lengths in the sc treated MPS VI rats (data not shown), as we had shown previously with oral PPS treatment [11].

### Effects of PPS Treatment on the Mobility of MPS VI Rats

**Figure 7:**
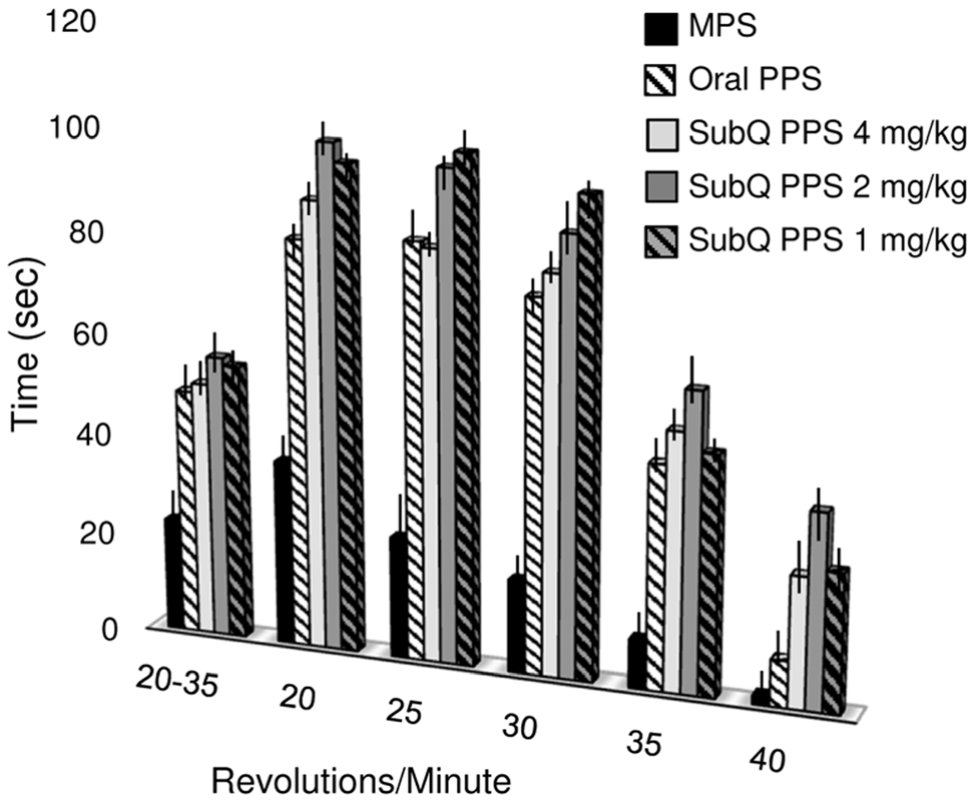
Motor activity in PPS treated MPS VI rats. Two weeks after the last PPS dose, treated MPS VI animals were subjected to rotarod analysis at five different speeds (range: 20–40 revolutions per min (RPM)), and their performance was compared to normal and untreated, age and gender-matched animals. Black columns represent untreated MPS VI rats; white hatched columns, oral 4 mg/kg PPS-treated MPS VI rats; light gray columns, 4 mg/kg sc PPS-treated MPS VI rats; dark gray columns, 2 mg/kg sc PPS-treated MPS VI rats; grey hatched columns, and 1 mg/kg sc PPS-treated MPS VI rats. All groups of treated MPS VI rats (oral and sc) remained on the rotating rod significantly longer than untreated animals (p<0.005). At the higher speeds (30, 35 and 40 RPM) MPS VI animals receiving all sc PPS doses had significantly better endurance and remained on the apparatus longer than those receiving oral PPS (p<0.05).

Fig. 7 summarizes rotarod analysis of the MPS VI rats at the end of the treatment period. All treatment groups exhibited significantly better rotarod performance than the age-matched, untreated MPS VI rats. This was increasingly evident at faster speeds. Also, at faster speeds a significant benefit of sc. vs. oral treatment was observed. Of note, the 4 mg/kg sc dose appeared to be less effective than 2 mg/kg. Videos 1 and 2 show representative 7-month-old untreated and treated (2 mg/kg, sc) MPS VI rats, respectively. Improved mobility and grooming is evident in the treated animal.

## Discussion

The rationale for investigating sc PPS treatment for MPS is based on the enhanced bioavailability of the injected compared to orally administered drug, as well as the potentially better compliance and cost-effectiveness of a once weekly injection versus daily administration in human patients. Further, for rat studies sc PPS injection permitted more consistent dosing than administration through the drinking water. Overall, the key benefits of PPS treatment on the MPS VI rats were the same or greater using sc vs. oral PPS. These common benefits included markedly improved mobility, improved tracheal morphology, increased trabecular bone in the femurs and vertebrae, improved dentition, including tooth mineral densities, and reduced levels of inflammatory markers in the serum. Neither mode of administration led to significantly longer femur lengths, although we did observe modest improvements in growth plate organization from sc treatment (Fig. 2). The shorter femur lengths in the MPS VI rats are most likely due to irregularities in the growth plate organization, and although we did observe modest improvements with sc PPS treatment, it appears this was not sufficient to promote the development of full length femurs.

In contrast to the femurs, we did observe positive effects on skull/snout lengths with both sc and oral treatment. It is notable that the bones in the jaw and mandibular area of the skull are the least mature bones in rats at 1 month of age (the age PPS treatment was initiated), and exhibit a linear increase in growth up to 6 weeks [24]. This, coupled with the fact that a majority of the bones in the skull form by intramembranous rather than endochondral ossification, may provide an explanation for the differences observed in femoral vs. skull lengths.

As noted above, microCT analysis revealed improvements in femoral and vertebral trabecular bone with PPS treatment. Very little effect was observed in cortical bone. More consistent trabecular effects were observed with sc vs. oral treatment, which may be attributed to more accurate dosing and biodistribution with injections compared to administration by gavage. It is known that some MPS patients have significantly lower bone mineral density (BMD) compared to normal [25], and this also was observed in the MPS VI rats. Subcutaneous PPS injection led to significantly increased BMD, and there was a dose dependent response. Trabecular bone is responsible for increasing the strength of bone when force is applied, which may also explain the enhanced strength observed from the 3-point bending tests (Fig. 5). In addition, performance of the treated MPS VI rats on an accelerating rotarod was greater using sc treatment, particularly at the faster speeds, which could be a direct effect of the aforementioned increased trabecular bone formation and flexural strength.

One of the surprising, positive outcomes from this study was that PPS treatment led to a reduction of GAG storage in the MPS VI rats. A notable difference between the oral and sc treatments was that in the latter significant reductions in tissue GAGs were observed. This might be explained by enhanced delivery of the drug to tissues following injection. To investigate this further, we used fluorescently labeled PPS and examined the tissue distribution by imaging 24 hours after single sc or oral administration (Fig. S3). Consistent with unpublished findings (personal communication – bene pharmaChem), we observed that liver and kidney were the major sites of PPS uptake, and that at 24 hours the amount of fluorescence detected in these tissues was significantly greater following sc injection compared to oral administration.

Since PPS is a low molecular weight, sulfated carbohydrate polymer that resembles GAG fragments, it is possible that once a critical level of the drug accumulates in tissues it might be interfering with GAG metabolism, either by slowing GAG synthesis or enhancing degradation by salvage pathways, thereby resulting in a reduction of GAG storage in the MPS animals. PPS also may have a direct effect on lysosomal function (e.g., correcting the autophagy defects), or perhaps bind to GAG-metabolizing enzymes (such as mutant *N*-acetyl galactosamine 4-sulfatase in the MPS VI rats), and have an enzyme enhancing/chaperone effect. Future studies will concentrate on the mechanism of PPS action in MPS, with an emphasis on gene/protein expression changes and alterations of GAG metabolic pathways.

Due to the enhanced tissue uptake and potential accumulation of PPS following sc injection, it was important to assess the long-term safety effects in the MPS animals. Throughout the 6-month study we monitored liver enzymes and various clotting factors weekly. There was no elevation of liver enzymes above baseline, and no abnormalities seen in clotting factor levels. No adverse effects of sc PPS treatment were observed. These results are consistent with our ongoing studies in MPS I dogs (unpublished results). The imaging studies with fluorescent PPS described above did reveal that after single sc administration fluorescence is retained in tissues for at least 2 weeks (data not shown), however it is unknown whether the fluorescent signal represents bioactive PPS or an inactive degradation product.

When considering the clinical utility of PPS in MPS patients, we predict that it may be used alone or in combination with other therapies, including ERT or HSCT. When used alone we anticipate that it will reduce inflammation systemically, as well as locally in joint tissues including the synovium and articular cartilage. This could result in reduced pain, increased mobility, and/or increased joint range of motion. Over time we also predict that it may slow the progression of tracheal deformities, enhance trabecular bone and bone strength, and perhaps result in reduced pulmonary infections and/or enhance lung function. Recent data obtained in MPS I dogs treated with sc PPS for 12 months also revealed markedly improved coronary artery disease compared to untreated MPS I animals (unpublished results).

One new biomarker we examined in the current study was S100A8/A9. The S100A8/A9 complex (also known as calgranulins or alarmins) is predominantly expressed and secreted by neutrophil granulocytes, and modulates inflammatory reactions by interacting with pattern recognition receptors, including the receptor of advanced glycation end products (RAGE), and TLR4 [26], [27]. Activation of the TLR4 pathway further up-regulates the expression of A100A8/A9, which in turn up-regulates MMPs, TNF-α, MCP-1 and IL-8 [28], [29]. In the current paper we show for the first time that A100A8/A9 and IL-8 are significantly elevated in serum from MPS VI animals. We have previously also shown increased AGEs in the MPS VI rats [11], which may be explained, at least in part, by elevation of the S100A8/A9 complex. A100A8/A9 also is elevated in rheumatoid arthritis and the levels correlate with disease severity and joint degradation [30]. Continued examination of the inflammatory pathways activated in MPS should identify additional biomarkers that could be used to monitor disease progression and treatment response, and further our understanding of the pathogenic mechanisms occurring in these complex disorders.

We previously showed that combining anti-TNF-α antibody therapy with ERT provided enhanced benefit over ERT alone, and anticipate a similar effect with PPS [12]. PPS has several advantages over anti-TNF-α antibody therapy, including the facts that it is more affordable and has an improved safety profile. Moreover, its mechanism of action in MPS may extend beyond its anti-TNF-α properties. ERTs are currently approved for four MPS types, and under development for several others [1], [31], [32]. However, the effectiveness of these therapies on the bone and joint manifestations of MPS is limited, and skeletal disease often continues to progress in treated patients [8], [10]. Similarly, HSCT also has limited effects on the bones and joints in MPS, and PPS could be a useful adjunct for transplanted patients as well [7]. Lastly, PPS has been administered into the CNS of patients with Creutzfeldt-Jakob disease, resulting in the reduction of neuroinflammation [33]. Since some MPS patients are currently receiving intrathecal enzyme infusions, and neurological MPS disease is characterized in part by neuroinflammation, these patients could similarly benefit from PPS treatment at the time of intrathecal enzyme administration [34], [35].

In conclusion, herein we show the benefits of sc vs. oral PPS administration in a rat model of MPS VI. The enhanced effects of sc PPS included a surprising and significant reduction in urine and tissue GAGs, as well as improvements in rotarod endurance, femoral and vertebral trabecular bone, and growth plate organization. Other benefits were common between the sc and oral treatment groups (e.g., reduction of tracheal deformities, improved dentition, reduction of serum inflammation markers). We also document the safety of weekly sc PPS administration in the MPS VI rats over a 6-month period. In the future, carefully controlled clinical trials should be carried out to evaluate the safety and efficacy of PPS in MPS patients, as well as mechanistic studies to investigate the nature of GAG reduction.

## Supporting Information

Figure S1 **Tracheal morphology.** Tracheas were collected from 7-month-old normal, untreated and PPS-treated MPS VI rats at the end of the study. As illustrated by this figure and similar to what was previously observed [11], untreated MPS VI rats had markedly abnormal, collapsed tracheas with narrow, flattened interior openings. These abnormalities were improved by sc PPS treatment in a dose dependent manner, resulting in rounded tracheas with almost normalized cross sectional areas.
(TIF)

Figure S2 **Femoral cortical microCT analysis.** Cortical values in all samples were variable and most of the measurements were not statistically different between normal or untreated MPS VI animals. The major cortical change observed was in porosity, which was higher in untreated MPS VI animals compared to normal, perhaps indicating an osteoporotic nature. Treatment with 1 and 2 mg/kg sc PPS appeared to normalize this value, but in the 4 mg/kg dose group it was significantly lower than normal.
(TIF)

Figure S3 **Biodistribution of labeled PPS in MPS VI rats.** PPS was labeled with Rhodamine B as described in Materials and Methods. MPS VI rats received a single administration of either oral or sc PPS at 10 mg/kg HED dose (n = 3/group). Treated and control MPS VI animals were sacrificed 24 hours post dose and kidney (**A–C**) and liver (**D–F**) were imaged. **A** and **D** show representative untreated MPS VI organs; **B** and **E** were from animals treated with oral PPS, while **C** and **F** were collected from animals receiving sc PPS. Fluorescence intensity in the tissues was greater following sc vs. oral PPS administration.
(TIF)

Movie S1 **Untreated 7 month old MPS VI rat.** Movie S1 shows the typical phenotype and appearance of an untreated adult MPS VI rat at 7 months of age. The rats display an ungroomed coat and a slow abnormal gait due to the progression of the disease.
(MOV)

Movie S2 **Treated 7 month old MPS VI rat at 2 mg/kg sc PPS dose.** Movie S2 shows the typical phenotype and appearance of an adult MPS VI rat at 7 months of age treated with 2 mg/kg sc PPS. The treated rats were capable of standing on their hind limbs and were much more active than the untreated littermates. Also, they had much smoother coats indicating the improved ability of grooming and much healthier gaits and appearances.
(MOV)
