# Supplementary figures and images for: Dose Responsive Effects of Subcutaneous Pentosan Polysulfate Injection in Mucopolysaccharidosis Type VI Rats and Comparison to Oral Treatment
Source: PLoS One. 2014 Jun 25;9(6):e100882. doi: 10.1371/journal.pone.0100882 (PMC4071040; doi:10.1371/journal.pone.0100882)

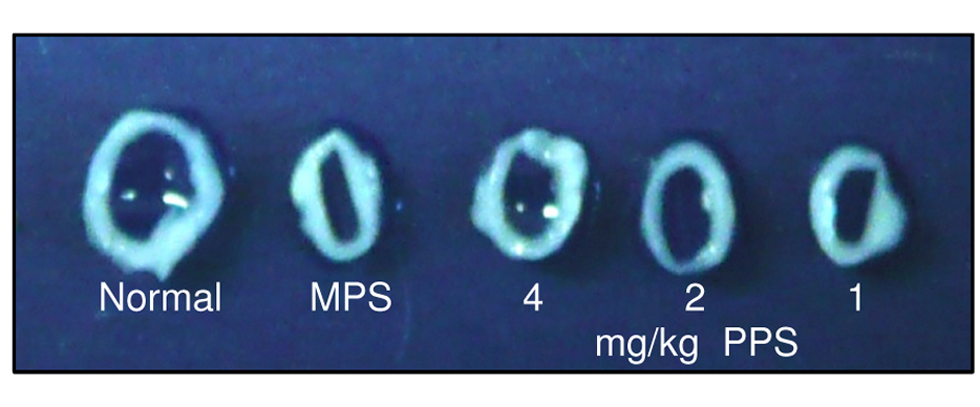

Supplement: Figure S1 — Tracheal morphology. Tracheas were collected from 7-month-old normal, untreated and PPS-treated MPS VI rats at the end of the study. As illustrated by this figure and similar to what was previously observed [11], untreated MPS VI rats had markedly abnormal, collapsed tracheas with narrow, flattened interior openings. These abnormalities were improved by sc PPS treatment in a dose dependent manner, resulting in rounded tracheas with almost normalized cross sectional areas. (TIF) [file pone.0100882.s001.tif]

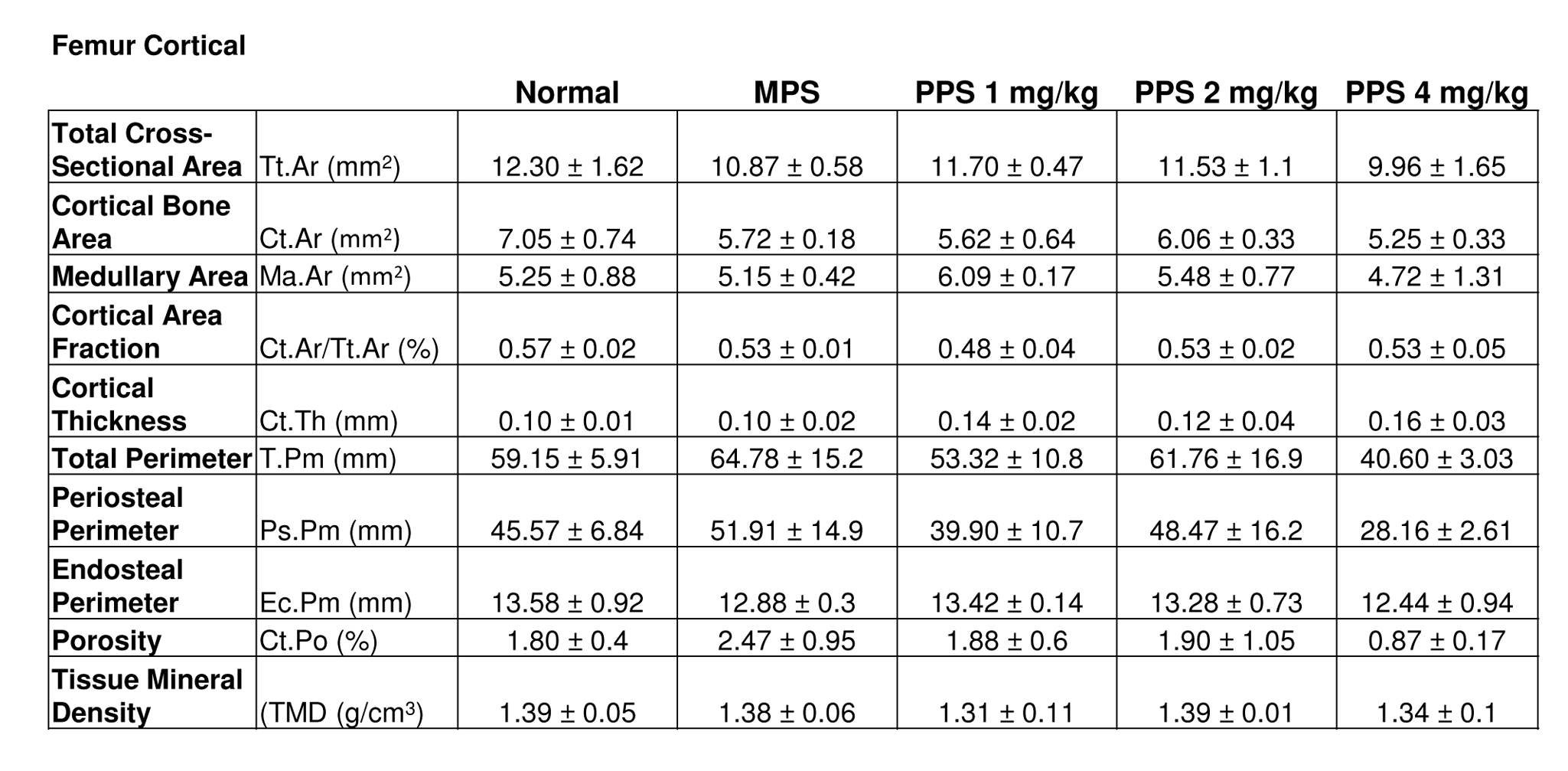

Supplement: Figure S2 — Femoral cortical microCT analysis. Cortical values in all samples were variable and most of the measurements were not statistically different between normal or untreated MPS VI animals. The major cortical change observed was in porosity, which was higher in untreated MPS VI animals compared to normal, perhaps indicating an osteoporotic nature. Treatment with 1 and 2 mg/kg sc PPS appeared to normalize this value, but in the 4 mg/kg dose group it was significantly lower than normal. (TIF) [file pone.0100882.s002.tif]

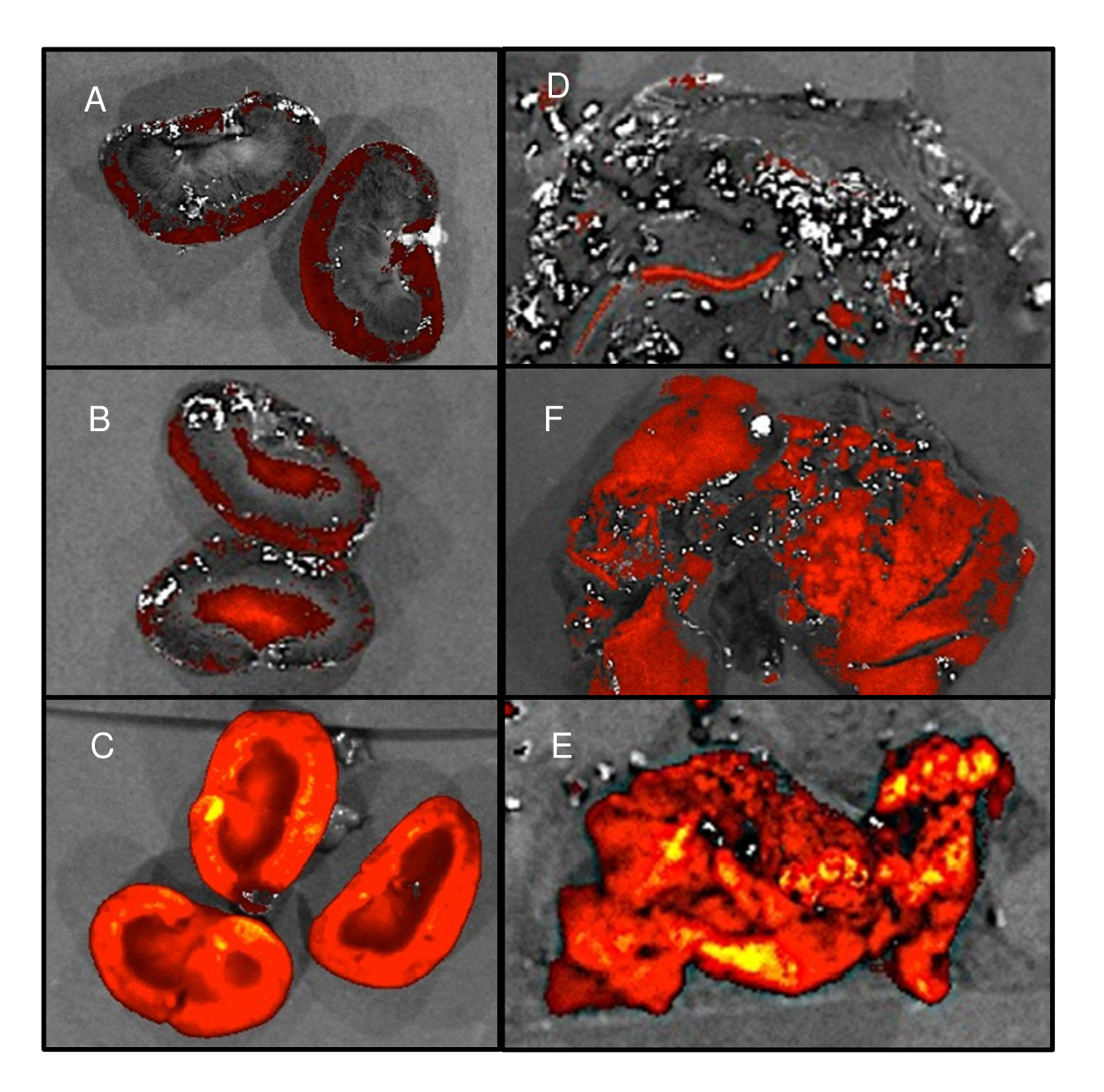

Supplement: Figure S3 — Biodistribution of labeled PPS in MPS VI rats. PPS was labeled with Rhodamine B as described in Materials and Methods. MPS VI rats received a single administration of either oral or sc PPS at 10 mg/kg HED dose (n = 3/group). Treated and control MPS VI animals were sacrificed 24 hours post dose and kidney (A–C) and liver (D–F) were imaged. A and D show representative untreated MPS VI organs; B and E were from animals treated with oral PPS, while C and F were collected from animals receiving sc PPS. Fluorescence intensity in the tissues was greater following sc vs. oral PPS administration. (TIF) [file pone.0100882.s003.tif]
